# Point-of-care ultrasound to inform antiviral treatment initiation in chronic hepatitis B virus infection in low-resource settings – the PUSH protocol

**DOI:** 10.1186/s13089-024-00369-2

**Published:** 2024-03-04

**Authors:** Tom Heller, Veronica Phiri, Tapiwa Kumwenda, Wongani Mzumara, Michael Jeffrey Vinikoor, Ethel Rambiki, Claudia Wallrauch

**Affiliations:** 1 Lighthouse Clinic Trust, Lilongwe, Malawi; 2 International Training and Education Center for Health, University of Washington, Seattle, WA USA; 3 Directorate of HIV/AIDS, STI and Viral Hepatitis, Ministry of Health, Lilongwe, Malawi; 4 Department of Internal Medicine, University of Zambia, Lusaka, Zambia; 5 University of Alabama at Birmingham, Birmingham, AL USA

**Keywords:** Ultrasound, Hepatitis B, Cirrhosis, HCC, Low-resource setting, Treatment indication

## Abstract

**Background:**

Chronic Hepatitis B (CHB) is prevalent worldwide and most related deaths occur in low-resource settings. Antiviral treatment of CHB is indicated in those with significant liver disease and markers of viral replication. However, recommended diagnostics such as elastography (a non-invasive imaging measure of fibrosis/cirrhosis) or HBV viral load are often lacking in these settings, which creates barriers to treatment. Point-of-care clinical B-mode ultrasound (US) has potential to overcome implementation barriers in HBV care programs in low-resource settings.

**Methods:**

We describe a Point-of-care US protocol for Hepatitis (“PUSH”) to check for signs of cirrhosis and hepatocellular carcinoma in the liver of people with CHB. We performed a prospective observational study applying the protocol, first by trainee clinicians and then by trainers, in consecutive patients referred to our clinic for CHB treatment eligibility assessment. All patients additionally underwent physical examination, liver function tests (LFTs) and platelet counts. We describe the PUSH training approach and performance of the protocol.

**Results:**

Four clinicians and 111 adult patients with HBV infection were included in the development of PUSH. Using US, liver complications of HBV were documented in 31 (27.9%) patients; including cirrhosis in 15 patients, HCC with cirrhosis in 13, and HCC without cirrhosis in 3. Patients with sonographic findings had significantly more clinical symptoms also their LFTs were higher and more frequently indicative for HBV treatment. Of 28 patients with sonographic diagnosis of cirrhosis, 23 (82.1%) showed a nodular liver surface, 24 (85.7%) a coarse echotexture, 20 (71.4%) scarce vessels, and 9 (32.1%) an enlarged caudate lobe. Overall concordance of the findings between assessment of trainees and experienced sonographers was high, ranging from 90 to 95%; trainees were not blinded to clinical and laboratory findings.

**Conclusion:**

Ultrasound can facilitate same-day initiation of antiviral therapy for chronic HBV monoinfection in a resource-limited setting and a streamlined protocol-driven liver ultrasound can be feasibly used by front line clinicians managing HBV.

**Supplementary Information:**

The online version contains supplementary material available at 10.1186/s13089-024-00369-2.

## Introduction

In 2019, 820.000 deaths related to hepatitis B virus (HBV) infection and more than 1.5 million new cases of chronic infection were reported worldwide [1]. The majority of deaths and new cases occurred in low-resource settings. In Malawi, hepatitis B surface antigen (HBsAg) seroprevalence among adults is estimated at 8.1% [2]. Unlike in HIV (i.e., universal treatment), treatment in chronic hepatitis B (CHB) is based on the degree of activity of the infection and treatment of CHB is only recommended when there is evidence of liver damage, such as fibrosis or cirrhosis, or of parameters (like viral load, family history) that predict future liver damage. Eligibility assessment in CHB is challenging to implement in low-income settings. HBV viral markers like HBV DNA, quantitative HBsAg, and HBeAg recommended by international guidelines [3, 4] are not included in standard laboratory package in most countries. If health facilities are unable to consistently guide people living with CHB on whether they need treatment, it can create missed opportunities to avert HBV-related deaths Therefore, innovative strategies to determine when to treat CHB in low-income settings are needed.

Ultrasound is a low-cost imaging modality that can identify chronic liver disease [5], and therefore should be strongly considered in HBV programs in resource-limited countries. It was estimated that in Sub-Saharan Africa between 4–13% of patients with CHB have cirrhosis [6] and although ultrasound is reported to have low sensitivity [7] especially for early cirrhosis, it can detect cases and thus provide therapy indication. While WHO guidelines suggest the use of ultrasound elastography as non-invasive fibrosis test [8], the use of B-mode ultrasound is not emphasized. B-mode ultrasound holds unique opportunities because of its potential reach: programs to train radiographers exist in nearly all low-resource countries, and most health facilities at secondary health care level and above have ultrasound machines and trained staff. Point-of-care ultrasound (POCUS) has extended the use of ultrasound from radiology departments to the bedside. An example in the African region is the “FASH protocol” (Focused assessment with sonography for HIV-associated TB [9]) for diagnosis of tuberculosis (TB) in people living with HIV, which has been widely adopted in HIV high-prevalence settings [10] and shown to have good specificity [11].

In Malawi’s new hepatitis guidelines [12] treatment eligibility is mainly based on ultrasound findings of cirrhosis or hepatocellular carcinoma (HCC) and on the aspartate-aminotransferase-to-platelet ratio index (APRI) for non-invasive assessment of liver fibrosis recommended by WHO [8] using a threshold of > 0.65 adapted for Sub-Saharan African settings [13]. We designed and evaluated a novel POCUS protocol to assess the liver of people with CHB mono-infection. Our goal was to develop a protocol that could be widely implemented by both radiographers and front-line clinicians, and help indicating therapy even in the absence of laboratory parameters. We describe the protocol, ultrasound findings in patients with CHB who presented to our center, and the feasibility of training clinicians to use the protocol with fidelity.

## Methods

### Point of care UltraSound for hepatitis (PUSH) protocol

**Fig. 1:**
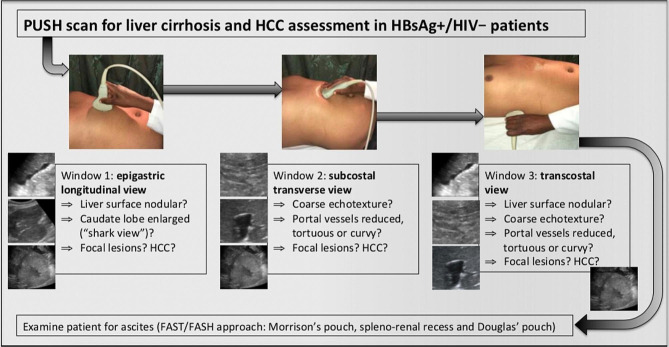
Point-of-care UltraSound for hepatitis - the PUSH protocol

The PUSH protocol assesses the liver using three sonographic windows to identify signs of liver cirrhosis. Four findings are looked for: (a) surface nodularity, (b) coarse echotexture, (c) changed vascularity and (d) enlarged caudate lobe. If any of the four findings is detected, alone or in combination, cirrhosis is considered present. A graphic summary of the protocol is shown in Fig. 1.

**Fig. 2:**
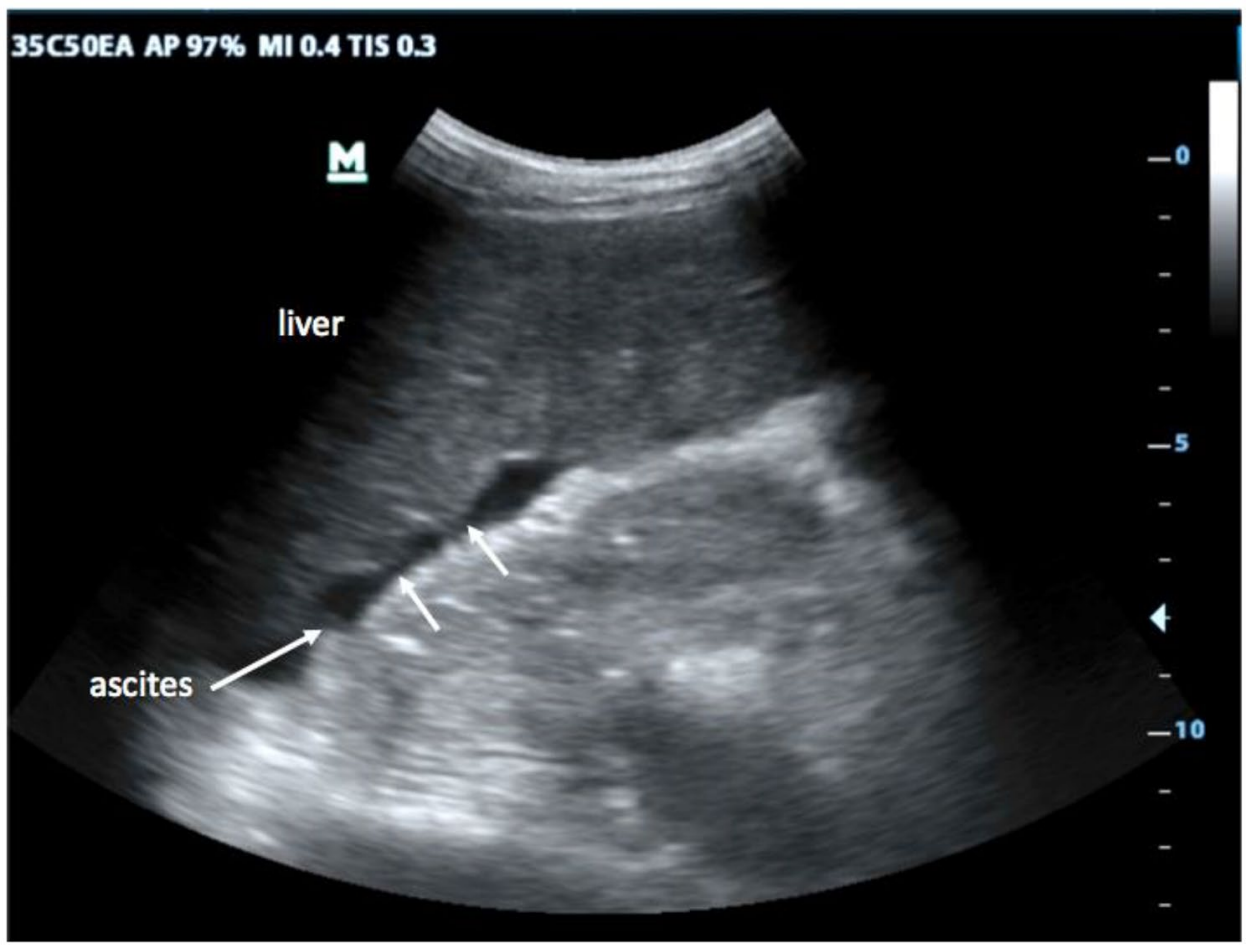
Surface nodularity: While normal liver shows a smooth, straight surface, it becomes irregular and nodular in cirrhosis due to regenerative cirrhotic nodules located in the subcapsular area (arrows). It is particularly obvious when ascites is present but can also be seen otherwise

**Fig. 3:**
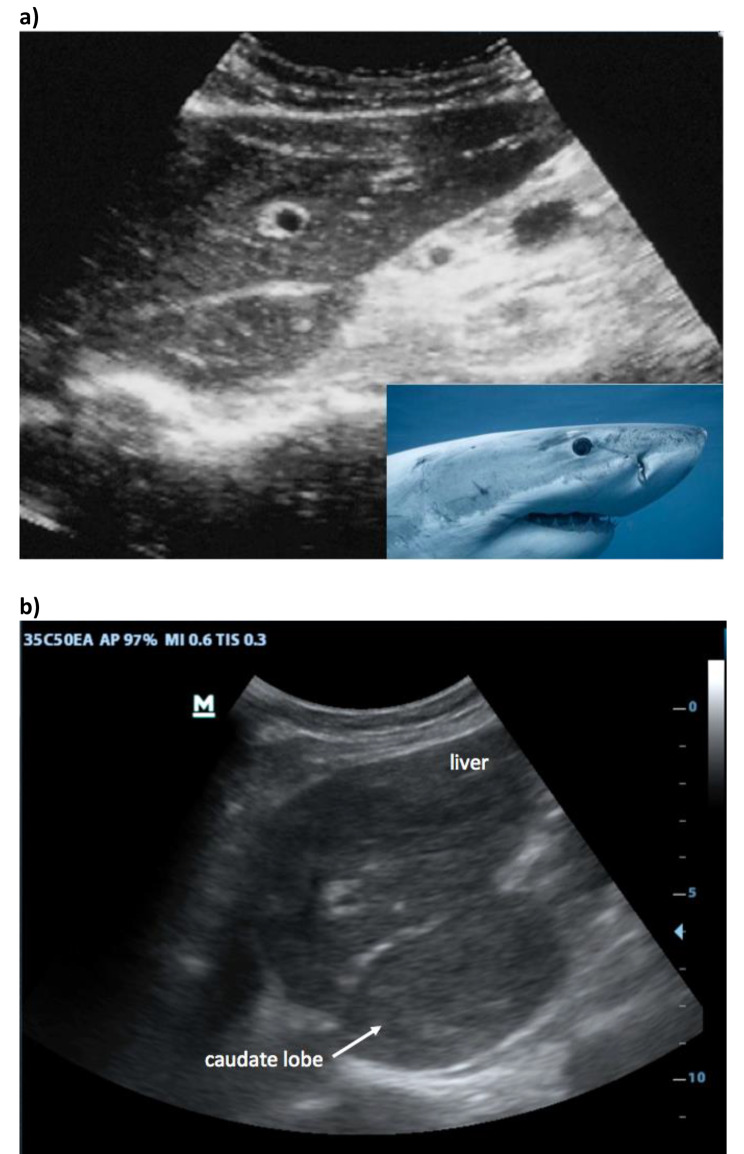
Enlarged caudate lobe: (**a**) In the normal liver the caudate lobe is small and in the epigastric longitudinal scan the profile resembles a shark. In cirrhosis, the caudate lobe may enlarge due to redistribution of blood supply and consecutive hypertrophy; the “shark face” is distorted (**b**)

**Fig. 4:**
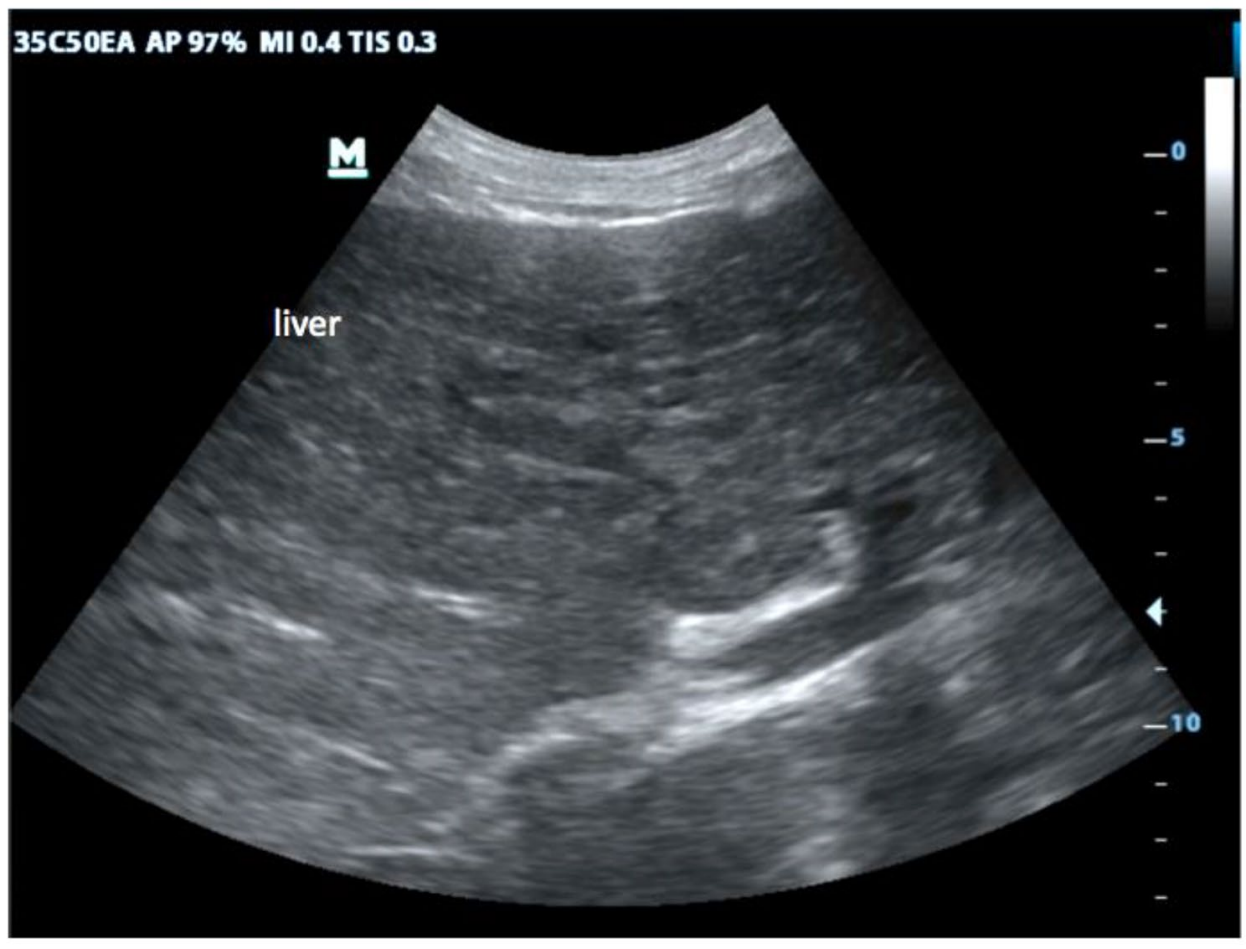
Coarse texture: While normal liver tissue shows a smooth, velvet-like texture in US this changes in cirrhosis. The formation of nodules, especially of macro-nodules (>3 mm) induced by CHB, causes a coarse, nodular texture throughout the liver

**Fig. 5:**
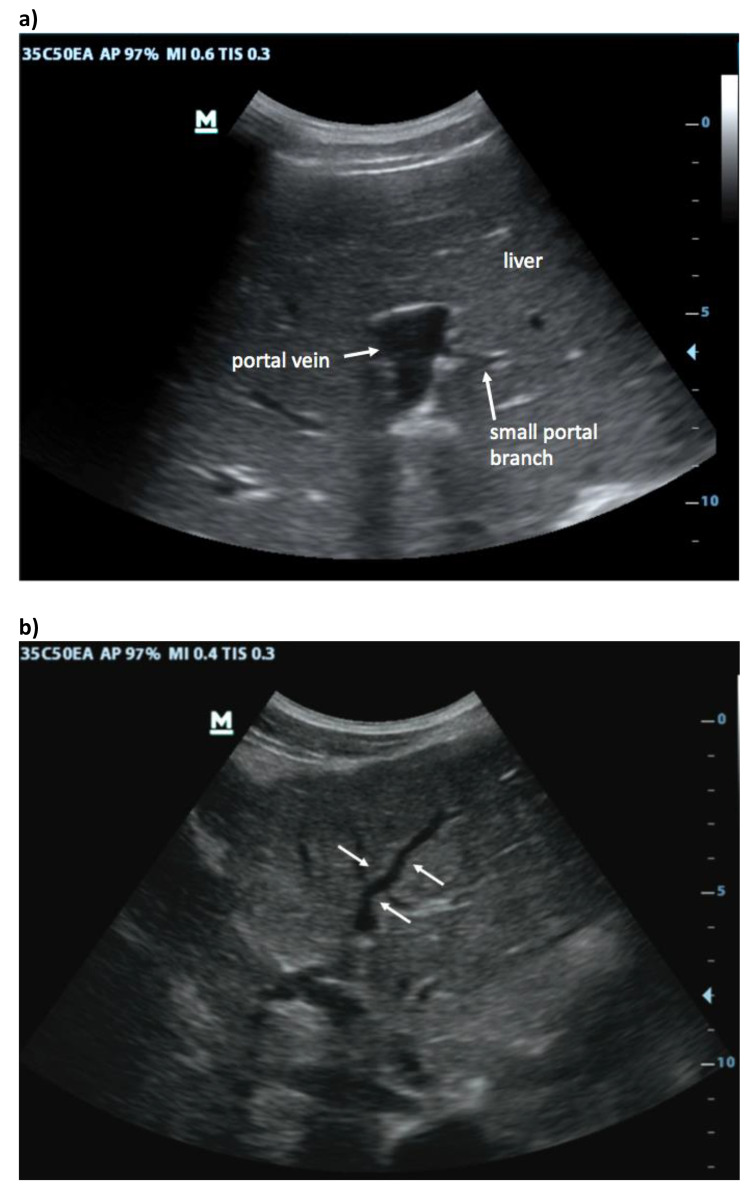
Reduced vascularity: (**a**) In the cirrhotic liver, the stiff tissue reduces the visibility of peripheral portal branches while at the same time the central portal vein may be enlarged due to portal hypertension. (**b**) Peripheral portal branches may show a tortuous course around regenerative nodules

**Fig. 6:**
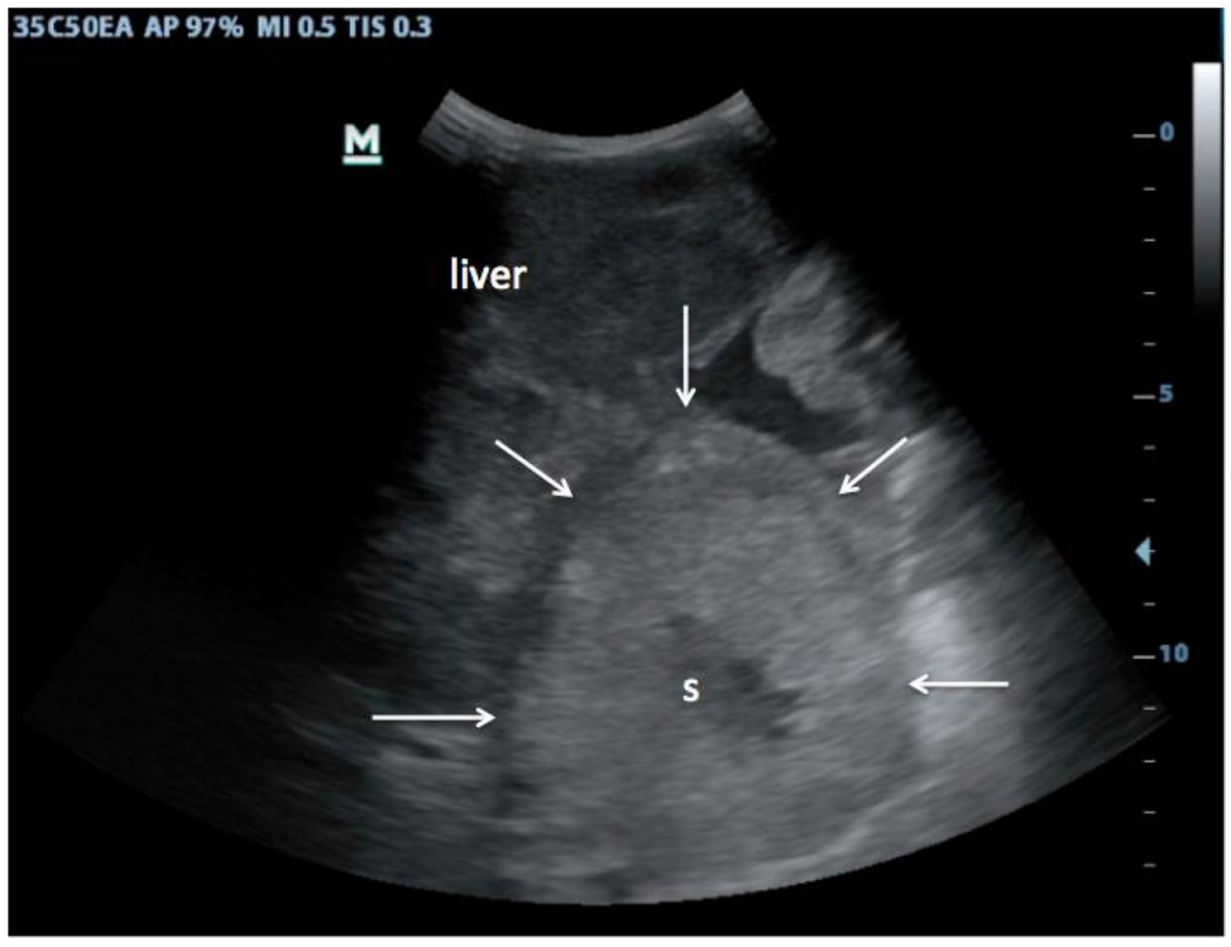
Hepatocellular carcinoma: Slightly hyperechoic, mosaic-like lesions which can show a hypoechoic halo and hypoechoic sinusoids in the tumor (s) as in this case are typical for HCC. In low-resource settings, HCCs are often large (~ 10 cm) at time of diagnosis

First, in the epigastric view, the surface is assessed for nodularity (Fig. 2) and size of caudate lobe (Fig. 3) is estimated by eyeballing the liver shape. Secondly, the subcostal view is used to assess the coarseness of echotexture of liver tissue (Fig. 4) and the visibility and course of portal vessels (Fig. 5). Third, the transcostal view, the liver is again assessed for surface nodularity, tissue texture and vascularity. Additionally, while performing the three sonographic windows, the operator should also look for the presence of focal lesions, which can be suggestive of HCC (Fig. 6). Due to late diagnosis of HCC in Malawi, many patients present with large, advanced tumors, which often have an echogenic mosaic appearance and may have a halo and central hypoechoic areas. Confirming the diagnosis of HCC is challenging in Malawi as other imaging modlities and alpha fetoprotein testing are not available.

After completing the three views, the final step in the protocol is assessment for the presence of ascites, which can provide evidence of decompensated cirrhosis. The well-established FASH approach is used, assessing for fluid in Morrison’s and Douglas’ pouch as well as in the spleno-renal recess [9]. The full protocol can be completed within few minutes.

### Pilot clinician training in PUSH

The PUSH protocol was both conceived of and piloted at Lighthouse Trust (LH), a WHO-recognized Center of Excellence for integrated HIV care operating multiple large HIV clinics in Malawi [14]. Its site at Kamuzu Central Hospital provides free outpatient HIV care for more than 13,000 patients on Antiretroviral Therapy (ART). As LH provides tenofovir as part of ART and prioritizes integration of other services into HIV care, it was chosen as an early implementer of the new hepatitis guidelines. At LH, staff routinely uses POCUS during HIV clinic visits to detect signs of disseminated TB, and multiple clinicians are trained and have 3 + years of experience with FASH using a portable ultrasound (DP-30, Mindray, China) with a convex probe (35C50EA). Beyond the regular use of POCUS, LH clinicians have no further formal ultrasound training.

To pilot the PUSH protocol, we trained LH clinicians caring for people with CHB and evaluated their performance of the protocol. As they were familiar with the basic concepts of ultrasound and the sonographic anatomy of abdomen (usually focusing on looking for signs of TB such as pathological fluids, enlarged lymph nodes, and spleen lesions), it was limited to liver anatomy and its pathological changes only. This was discussed using a 2-hour classroom session with lecture with video material (supp. material 1). A job aid (supp. material 2) summarizing the pathological changes to the liver seen in CHB was created, printed and placed adjacent to the ultrasound for easy access. After this, clinicians incorporated the protocol with their routine work-up of CHB treatment eligibility. For a four-week period, patients with CHB underwent ultrasound by a trainee clinician using the PUSH protocol supervised by a trainer (CW and TH, two internal medicine specialists with longstanding ultrasound experience in resource-limited settings). After this period, trainee clinician independently documented the findings on a clinical report form (CRF). The patients and recorded clips were afterwards reviewed and discussed with the trainers, who were working nearby, and the patient was scanned again if this was deemed necessary. During a short period, review was only possible remotely by sharing the clips and the clinical information electronically. CRFs had a section for results of the trainer assessment to be documented, allowing estimating concordance of the trainee and trainer assessments.

### Statistical data analysis

Data were retrospectively extracted from patient files, anonymized and collected in a protected file. Statistics were calculated using Excel and MedCalc’s online calculators [15]. Concordance was estimated as proportion of “correct” POCUS results compared to the trainer POCUS assessment. Inter-rater agreement was assessed calculating Cohen’s Kappa statistic [16] and was interpreted as fair agreement (0.21–0.40), moderate (0.41–0.60), substantial (0.61–0.80) and near perfect (0.81–1.00). All investigations and data collection were part of routine clinical care and processes at LH; approval was granted by the Malawi National Health Science Research Committee for the collection and use of clinical and programmatic data (NHSRC Protocol #2812) used in this report.

## Results

### Cohort description

From January to July 2023, feasibility of the protocol was evaluated in 111 patients with CHB who presented for a treatment eligibility visit. The median age of patients was 31 years (IQR 28–37), 72 (64.8%) were male, and 39 (35.1%) were female. HBV-related liver complications were documented in 31 (27.9%) patients. Cirrhosis was detected in 28 patients (25.2%); in 13 (46.4%) of them lesions suspicious for HCC were seen. HCC without cirrhosis were detected in additional three (2.7%) patients. In 12/31 (38.7%) ascites was documented sonographically. Characteristics of patients with and without liver abnormalities on ultrasound are shown in Table 1. In patients with abnormalities, APRI score was more often above the threshold of > 0.65 (17/31 [54.8%]) than in those with a normal liver on ultrasound (14/80 [17.5%]; *p* = 0.001).

**Table 1:** Patient characteristics of patients referred to LH clinic for assessment for antiviral therapy of chronic hepatitis B

	Patients with ultrasound abnormalities (*n* = 31)	Patient without ultrasound abnormalities (*n* = 80)	
**Demographic**			
Sex (M/F, %)	25/6 (80.6%/19.4%)	47/33 (58.7%/41.2%)	*p* = 0.0309
Age, years [IQR]	35 [30–40]	30 [28–36]	
**Signs/symptoms**			
Abdominal swelling (%)	19 (61.3%)	5 (6.3%)	*p* < 0.0001
Abdominal pain (%)	18 (58.1%)	6 (7.5%)	*p* < 0.0001
Jaundice (%)	7 (22.6%)	8 (10.0%)	n.s.
Nausea/Vomiting (%)	11 (35.5%)	7 (8.8%)	*p* = 0.0007
**Laboratory results**			
ALT (mean, 95% CI)	70 [50–91]	136 [57–214]	n.s.
AST (mean, 95% CI)	211 [117–305]	95 [43–146]	*p* = 0.024
Bili_tot_ (mean, 95% CI)	3.6 [1.5–5.8]	2.6 [1.0-4.2]	n.s.
APRI^#^ (mean, 95% CI)	5.0 [1.4–8.5]	1.6 [0.7–2.6]	*p* = 0.012
**HBV Treatment**			
Treated	31 (100%)	14 (17.5%)	*p* < 0.0001

#APRI Aspartase-aminotransferase (AST)-to-platelet ratio index calculated as (AST/AST-ULN) / platelet count x 100

According to Malawi guidelines the APRI score should not be used for patients with very high AST or ALT levels (≥ 300 U/l) as these levels may be more indicative of acute than chronic hepatitis B. 10 (9.0%) patients had such high APRI scores. In five of them, tenofovir-based therapy was nevertheless initiated after the PUSH ultrasound revealed signs of cirrhosis and/or HCC. In the remaining five the ultrasound was normal and patients were monitored off-therapy with serial LFTs. Over the following weeks, LFTs normalized suggesting resolution of acute hepatitis.

### Comparison of eligibility for therapy by ultrasound and APRI score

Overall 45 (40.5%) of the 111 patients were eligible for antiviral treatment of CHB based on the comprehensive assessment (laboratory and ultrasound). Treatment indication was based on an elevated APRI score alone in 14 (31.1%) patients and on pathological ultrasound findings alone in 14 (31.3%); 17 patients (37.8%) met both criteria.

### Frequency of individual ultrasound findings

Of the 28 patients with the final sonographic diagnosis of cirrhosis (with or without HCC) 23 (82.1%) showed a nodular surface, 24 (85.7%) a coarse echotexture, 20 (71.4%) scarce vessels, and 9 (32.1%) an enlarged caudate lobe. One patient had only a nodular liver surface and another one had only coarse echo texture; 26 of 28 patients with cirrhosis had two or more concomitant findings. Seven of 28 (25.0%) patients showed all four findings. All patients with enlarged caudate lobe had also other findings that would have led to the diagnosis of cirrhosis. Focal liver lesions suspicious for HCC were seen in 16 patients; the lesions were seen in a cirrhotic liver in 13 (81.2%) and in a normal liver in 3 (18.8%). Ascites was detected in 5/15 (33.3%) patients with cirrhosis, 1/3 (33.3%) with HCC only, and 6/13 (46.1%) of patients with cirrhosis and HCC.

### Concordance of trainee scans compared to expert scans

In a subgroup of 70 participants the trainee initially scanned patients and an expert afterwards reviewed patient and scan. Four trainees scanned 32, 20, 10 and 8 patients respectively. In 64 (91.4%) direct patient review at the ultrasound machine was done, in 6 cases (8.6%) clinical information and video clips were sent electronically. Presence or absence of a nodular surface was correctly identified in 67 (95.6%; 2 were missed, 1 was over-diagnosed, Cohen’s kappa κ = 0.89); coarse echotexture was correctly ascertained in 66 (94.2%; missed in 2, over-diagnosed in 2 cases, κ = 0.86). Scarcity of vessels was correctly classified in in 64 (91.4%; missed in 4, over-diagnosed in 2 cases, κ = 0.76) and enlarged caudate lobe size in 63 (90.0%; missed in 6, over-diagnosed in 1 cases, κ = 0.58). In the overall assessment, the trainee concluded “no cirrhosis” in three cases while expert review later diagnosed cirrhosis (κ = 0.89). For the diagnosis of HCC, there was discordance in three cases; in these cases the trainee missed the suspicious lesion (κ = 0.84).

## Discussion

In this report, we demonstrate that in an integrated HIV-HBV clinic in Malawi 25.2% of referred patients had sonographic signs of cirrhosis. Overall, treatment was indicated in 45 patients and POCUS alone can help to indicate same-day initiation of HBV antiviral therapy in 68.9% of them. It was further found that clinicians can be trained to provide liver POCUS with fidelity.

Changes suggesting liver cirrhosis well described in the literature are irregular liver surface [5, 17, 18] (Fig. 2), coarse echotexture of the tissue [5, 17, 18] (Fig. 4) as well as paucity of peripheral vessels, which may have a tortuous course [17] (Fig. 5). Liver surface nodularity is considered likely the most sensitive and reproducible US signs of cirrhosis [19, 20]. It has been suggested [21, 22] that the use of a linear high-frequency probe can increase the sensitivity of liver ultrasound, especially in detecting surface changes and echotexture. While this was not used during our study, we have since found it helpful for assessment of ambiguous cases. In line with previous reports [23], we noted that findings of cirrhosis due to HBV may be easier to visualize than those due to other causes, as CHB usually leads to macro-nodular cirrhosis [24]. This leads to more marked changes and all sonographic findings were frequently seen in our patient population.

Changes of the liver shape in cirrhosis are also well described [17, 18] with shrinking of the right lobe and relative enlargement of the left and especially of the caudate lobe. It is attributed to relative obstruction of the hepatic veins, causing greater blood flow through the caudate lobe and thereby hypertrophy [25]. In our protocol, we did not use the measured comparison to the right liver lobe originally proposed [26], but instead eyeballed its size using the liver shape in the sagittal view [17] (Fig. 3). Detection of more subtle anatomical changes in cirrhotic livers, like expansion of the gallbladder fossa [27] and enlarged hilar space [28], were not included in the protocol. In our experience, the coarse echotexture (85.7%) and the nodular surface (83.1%) were most frequently detected followed by scarcity of vessels (71.4%) and were comparable in their diagnostic value. An enlarged caudate lobe was only recognized in 32.1% of our patients and was always associated with other changes, so it did not add to diagnostic accuracy and could potentially be dropped from the protocol.

Many patients with HCCs present late in our setting and have large palpable tumors, sometimes visible as an abdominal bulge [29], which makes their sonographic detection easier (Fig. 6). Ultrasound findings described of advanced HCC are a mosaic pattern, which is often hyperechoic and may have peripheral halo [30]. Dilated intra-tumoral blood sinusoids and perinodular daughter nodule formation are seen. It should be noted that unlike in chronic HCV and other chronic liver diseases, HCC can occur in the context of CHB without cirrhosis, and is an especially featured cancer in young male African patients with CHB [31].

As discussed, while ultrasound is useful to identify cirrhosis, it is not sufficiently sensitive in detecting early stages of liver fibrosis [7, 32]. It is therefore important to use other approaches of non-invasive liver assessment in parallel like e.g., the APRI score with an adapted threshold [13]. Ultrasound elastography is recommendable [3, 4, 8] and would be helpful if simple, stable and affordable devices were available. These gaps in assays for liver disease staging and absent HBV markers like viral load are expected to undermine scale-up of HBV therapy; therefore, a liver POCUS protocol with even moderate sensitivity may help close gaps in the care cascade.

The feasibility to train clinicians in a POCUS approach to the cirrhotic liver and HCC is essential for a wider utilization of ultrasound in the resource-poor setting as radiological services are often scarce [33]. The agreement in our study between trainer and trainee showed substantial or near perfect agreement for all findings except for assessment of caudate lobe size, where only moderate agreement (κ = 0.58) was seen. These values suggest that trainees can recognize the changes with fidelity. A recent study in a high-resource setting showed that emergency physicians can significantly increase their sensitivity and specificity in diagnosing cirrhosis using ultrasound [34]. The study assessed only image interpretation but not image acquisition; nevertheless it convincingly argues, that viewing the liver is frequently required in protocols like the FAST exam, and clinician thus should have competency to obtain images of liver parenchyma and surface. Another recent study favorably compared hand-held POCUS by clinical hepatologists to radiology ultrasound, transient elastography, and liver biopsy [35].

Our study has a few weaknesses that warrant discussion. Our trainee clinicians were previously trained in POCUS, especially in FAST and FASH [9] but also e.g., in cardiac applications [36]. These are frequently used, so they cannot be described as “inexperienced” in clinical ultrasound. Therefore, the feasibility to train clinicians with less POCUS experience will still need to be established. Another limitation was that, in our evaluation, trainee clinicians were not blinded to the clinical and the laboratory results. Furthermore, they were able to discuss scans between each other as part of their clinical routine. Therefore, they may have reported ultrasound findings of cirrhosis based on taking a history, physical examination, and/or review of lab values. Feedback by the expert was given on each case thus improving their learning curve over time. The high concordance between the scans should thus not be over-interpreted. In further studies, the PUSH protocol should be validated in a blinded study design, i.e., without US operators being aware of the laboratory results as these could influence the interpretation of ultrasound findings. Additionally, future evaluations should possibly include comparison to other modalities (e.g., elastography). Nevertheless, our pilot data shows that liver ultrasound using a simple, standardized approach like the PUSH protocol can be integrated in clinical care in resource-poor settings when ultrasound is available and training can be provided.

## Conclusion

Ultrasound can facilitate same-day initiation of antiviral therapy for CHB mono-infection in a resource-limited setting and a streamlined liver POCUS can be feasibly used by front line clinicians managing HBV.

### Electronic supplementary material

Below is the link to the electronic supplementary material.

Supplementary Material 1

Supplementary Material 2

## Data Availability

Not applicable.

## Abbreviations

95% CI: 95% confidence interval
ALT: Alanine aminotransferase
ART: Antiretroviral Therapy
APRI: Aspartate aminotransferase-to-platelet ratio index
AST: Aspartate aminotransferase
CD4: Cluster of differentiation 4
CHB: Chronic hepatitis B
CRF: Clinical report form
HBV: Hepatitis B Virus
HBsAg: Hepatits B surface antigen
HCC: Hepatocellular carcinoma
HIV: Human immunodeficiency virus
FASH: Focused Assessment with Sonography for HIV-associated TB
FAST: Focused Assessment with Sonography for Trauma
IQR: Interquartile range
n.s.: Not significant
POCUS: Point of care ultrasound
PUSH: Point-of-care US for Hepatitis
TB: Tuberculosis
US: Ultrasound
WHO: World Health Organization
