# Supplementary material for: Point-of-care ultrasound to inform antiviral treatment initiation in chronic hepatitis B virus infection in low-resource settings – the PUSH protocol
Source: Ultrasound J. 2024 Mar 4;16:18. doi: 10.1186/s13089-024-00369-2 (PMC10912069; doi:10.1186/s13089-024-00369-2)
Supplement: Supplementary file 1 — Supplementary Material 1 [file 13089_2024_369_MOESM1_ESM.pdf]

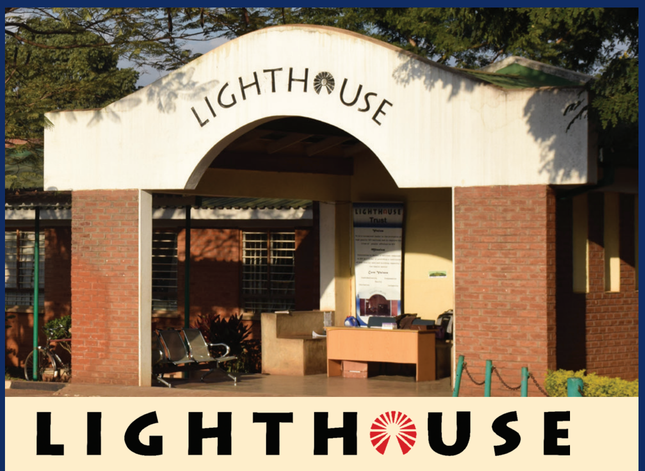

Liver Ultrasound for HBsAg-Positive Clients: Cirrhosis & Hepatocellular Carcinoma (HCC)

Normal Liver

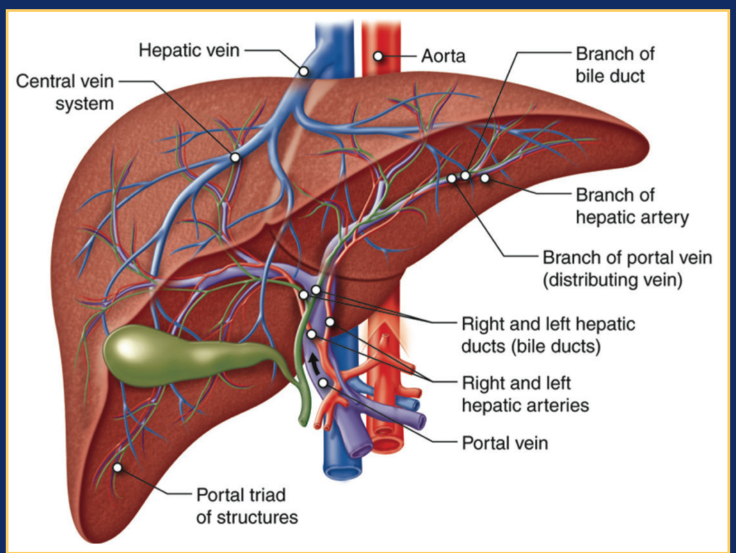

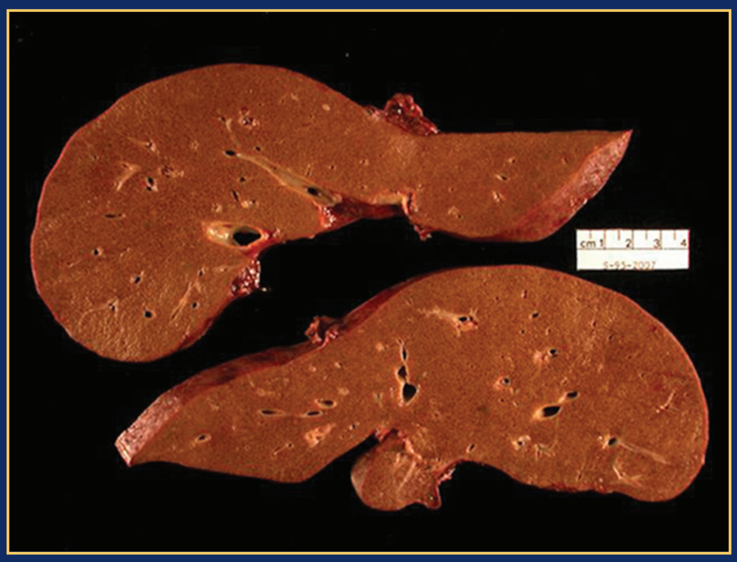

Portal and **hepatic veins** branch throughout the liver. (Intrahepatic bile ducts are not visible on ultrasound.) The liver tissue has a smooth, **velvet-like echo texture** and a **smooth surface**, as in the central image below.

Scan Windows for the Liver

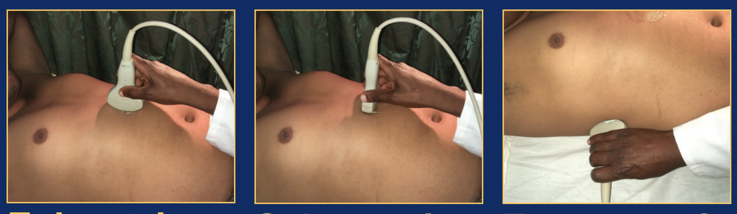

Epigastric longitudinal | Subcostal transverse | Transcostal

Cirrhosis

Liver tissue is replaced by **regenerative, fibrotic nodules**. Important ultrasound findings are:

1) **Irregular surface** due to bulging of the nodules—best seen when ascites is present
2) Small **vessels meandering** around the nodules, often even completely 'compressed'—**amputated and rarefied**
3) **Coarse, mottled echo pattern** of the liver tissue
4) **Enlarged caudate lobe** and shrinking of the right liver lobe (due to changes in blood perfusion)

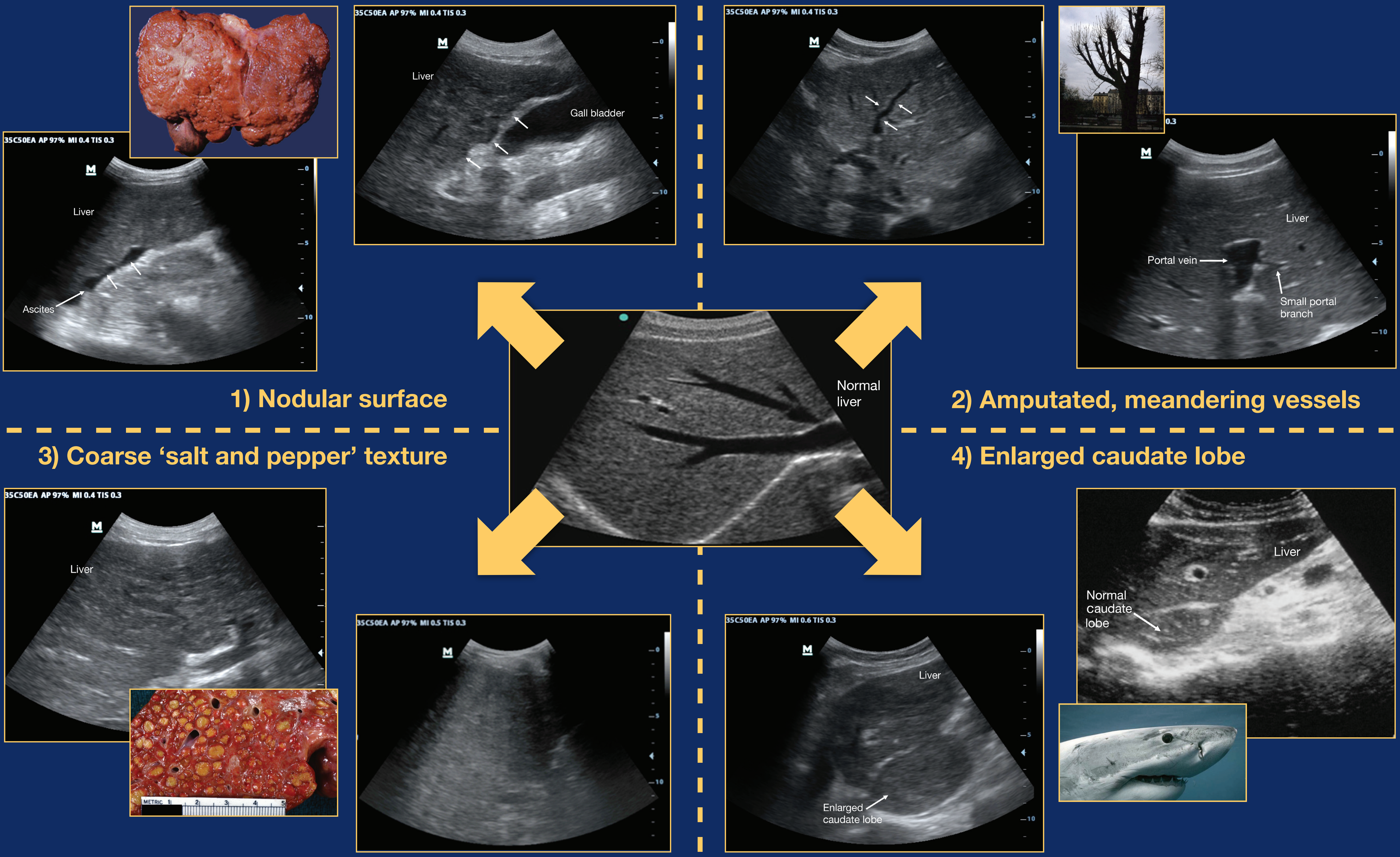

HCC

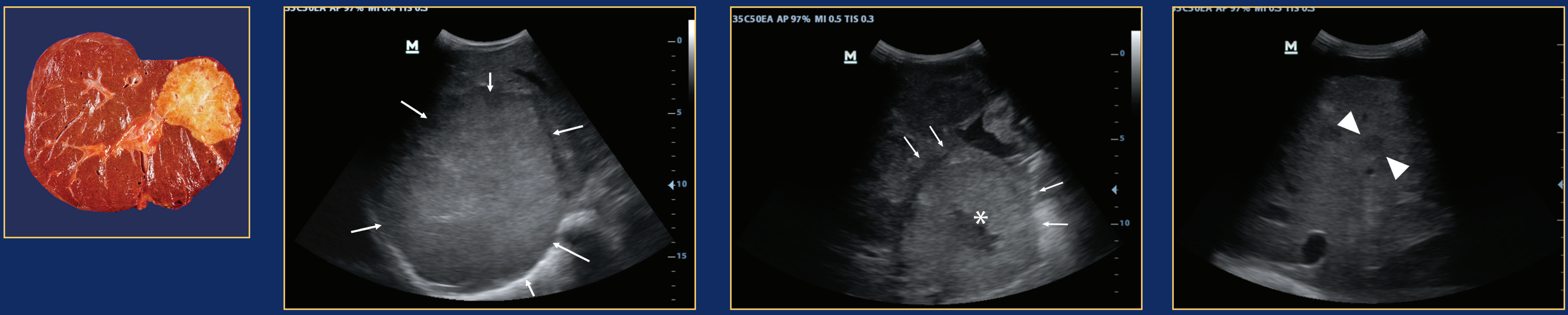

Often **single** large **liver tumours** with mixed echogenecity, resulting in a **'mosaic pattern'**. You may see a **hypoechoic halo** (arrows) and **necrotic central areas** (*). Small (<2 cm) HCCs are **hypoechoic nodules** (arrowheads). HCC is **often seen in cirrhotic livers**, but **also** with hepatitis B infection in normal livers.

LIGHTHOUSE LIGHTHOUSE
